# Clinical Profile of Dementia in Bed‐Bound or Home‐Bound Elderly Individuals from Low‐Income Communities in Southeast Brazil

**DOI:** 10.1002/alz.093336

**Published:** 2025-01-09

**Authors:** Ana Paula Bernardes Real, Flavia Lanna de Moraes, Edgar Nunes de Moraes, Carla Jorge Machado

**Affiliations:** ^1^ Jenny de Andrade Faria Institute – Reference Center for the Elderly, Federal University of Minas Gerais (UFMG), Belo Horizonte, Brazil, Belo Horizonte, Minas Gerais Brazil; ^2^ Cog‐Aging Research Group, Universidade Federal de Minas Gerais (UFMG), Belo Horizonte, Minas Gerais Brazil; ^3^ Department of Internal Medicine, School of Medicine, Federal University of Minas gerais, Belo Horizonte, Minas Gerais Brazil; ^4^ Federal University of Minas Gerais, Belo Horizonte, Minas Gerais Brazil; ^5^ Geriatrics and Gerontology Center Hospital of University of Minas Gerais, Belo Horizonte, Minas Gerais Brazil; ^6^ Faculty of Medicine, Federal University of Minas Gerais (UFMG), Belo Horizonte, Minas Gerais Brazil

## Abstract

**Background:**

Aging is linked to an increased risk of dementia, a significant cause of functional decline in the elderly, necessitating long‐term care.

**Methods:**

Dementia prevalence rates by age categories were obtained for bed‐bound or home‐bound elderly individuals from a public health system’s home care service in communities of Belo Horizonte/MG, to assess the independent effect of age. Analyses were conducted using Stata/SE for Macintosh 12.0 and included descriptive measures and adjusted prevalence ratios using Poisson Regression analysis with robust standard deviations. Results were considered significant at p<0.05.

**Results:**

A total of 1,092 dementia patients were evaluated, aged 70‐79 (28.6%), 80‐84 (19.9%), and 85‐90 (18.9%). Over half had less than 4 years of education (53.5%). The primary etiologies of dementia were Alzheimer’s disease alone (28%), isolated vascular dementia (13.8%), and mixed dementia (10.9%). The prevalence of other etiologies like frontotemporal dementia, Lewy body dementia, and dementia associated with Parkinson’s disease were low. Dementia prevalence by age group was 39.4% (60‐69 years); 55.6% (70‐79); 58.3% (80‐84); 69.6% (85‐89); 72.2% (90‐94) and 79.1% (95 years or older). Compared to the age group 60‐69 years, the PR was 1.4 for 70‐79; 1.5 for 80‐84; 1.8 for 85‐89; 1.9 for 90‐94; and 2.1 for 95 years or older (p<0.005 for all PRs). The average Charlson Comorbidity Index (CCI) was 5.1 (standard deviation 1.8), and the proportion of frail elderly was 95%. Poisson regression revealed that dementia prevalence was not affected by CCI or education level, but there was an independent effect of age group and frailty. Male gender was negatively and independently associated with dementia (PR = 0.80; p<0.001), while frailty was positively and independently associated with dementia (PR = 14.9; p<0.001).

**Conclusion:**

The primary cause of dementia was Alzheimer’s disease, following the pattern reported in the literature. Age was independently associated with dementia. Dementia was strongly linked to the severity of the clinical‐functional frailty presented by the elderly.

